# Silhouette width using generalized mean—A flexible method for assessing clustering efficiency

**DOI:** 10.1002/ece3.5774

**Published:** 2019-11-19

**Authors:** Attila Lengyel, Zoltán Botta‐Dukát

**Affiliations:** ^1^ Department of Vegetation Ecology University of Wrocław Wrocław Poland; ^2^ Institute of Ecology and Botany Centre for Ecological Research Vácrátót Hungary

**Keywords:** cluster validation, clustering, compactness, connectedness, generalized mean, separation, silhouette width

## Abstract

Cluster analysis plays vital role in pattern recognition in several fields of science. Silhouette width is a widely used index for assessing the fit of individual objects in the classification, as well as the quality of clusters and the entire classification. Silhouette combines two clustering criteria, compactness and separation, which imply that spherical cluster shapes are preferred over others—a property that can be seen as a disadvantage in the presence of complex, nonspherical clusters, which is common in real situations. We suggest a generalization of the silhouette width using the generalized mean. By changing the *p* parameter of the generalized mean between −∞ and +∞, several specific summary statistics, including the minimum, maximum, the arithmetic, harmonic, and geometric means, can be reproduced. Implementing the generalized mean in the calculation of silhouette width allows for changing the sensitivity of the index to compactness versus connectedness. With higher sensitivity to connectedness, the preference of silhouette width toward spherical clusters should reduce. We test the performance of the generalized silhouette width on artificial data sets and on the Iris data set. We examine how classifications with different numbers of clusters prepared by different algorithms are evaluated, if *p* is set to different values. When *p* was negative, well‐separated clusters achieved high silhouette widths despite their elongated or circular shapes. Positive values of *p* increased the importance of compactness; hence, the preference toward spherical clusters became even more detectable. With low *p*, single linkage clustering was deemed the most efficient clustering method, while with higher parameter values the performance of group average, complete linkage, and beta flexible with beta = −0.25 seemed better. The generalized silhouette allows for adjusting the contribution of compactness and connectedness criteria, thus avoiding underestimation of clustering efficiency in the presence of clusters with high internal heterogeneity.

## Graphical abstract

Silhouette width is widely used for assessing clustering efficiency in various scientific fields including molecular biology, ecology, and taxonomy. We present a generalization of this index which allows for adjusting its sensitivity to cluster compactness and connectedness. With the proposed flexible formula, it is possible to evaluate clusters of different shape, size, and internal variation more reliably.

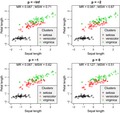

## 1 INTRODUCTION

Cluster analysis is the method of grouping similar objects in order to simplify the structure of a data set. It is concerned with discontinuous variation in the data set that allows for separating and identifying “types” of objects. Clustering is a common exploratory tool for pattern recognition in large samples in various fields of science, like geoinformatics (e.g., Lu, Coops, & Hermosilla, 2016), genomics (e.g., Ramoni, Sebastiani, & Kohane, 2002), epidemiology (e.g., Kenyon, Buyze, & Colebunders, 2014), or psychology (e.g., Clatworthy, Buick, Hankins, Weinman, & Horne, 2005). Moreover, classification is a prerequisite for naming abstract entities like biogeographical regions and habitat types; thus, it is a basic statistical approach in bioregionalization (e.g., González‐Orozco, Laffan, Knerr, Miller, & Jetz, 2013; Lechner et al., 2016) and vegetation typology on different scales (e.g., De Cáceres et al., 2015; Lengyel et al., 2016; Marcenò et al., 2018). Clustering methods could be divided into two groups according to three independent aspects: (a) crisp versus fuzzy clustering, (b) hierarchical versus nonhierarchical clustering, and (c) model‐based versus heuristic algorithms. Crisp clustering procedures provide unequivocal assignment of objects to groups, while fuzzy methods express degrees of membership as weights and allow for assigning an object to multiple groups at a time. The advantage of fuzzy classification over crisp methods is that they enable differentiation of typical, transitional, and outlier objects (De Cáceres, Font, & Oliva, 2010). However, fuzzy algorithms are much more intensive computationally and they require more subjective decisions from the user for the parameterization; therefore, crisp methods are still the most widespread. Hierarchical methods classify the objects into groups which are nested subsets of each other, while nonhierarchical methods produce a simple partition without nested structure. Model‐based clustering fits mixture of distributions on the observed data optimizing the likelihood function, while heuristic methods optimize different other (most often geometric) criteria. Despite the fact that fuzzy classification and hierarchical methods offer additional information, the most common objective of numerical classification is to group the objects into mutually exclusive, exhaustive sets, that is, to produce a partition. In spite of advantages of model‐based methods, partitions are often created by heuristic methods. Its reasons are that (a) model‐based methods are much more computation intensive that limits their application in large datasets; (b) data do not always follow a simple distribution type or there is no reasonable a priori information on the distribution; and finally (c), there may be cluster shapes that are hardly captured by fitting simple mixtures.

By its basically descriptive nature, clustering techniques, especially crisp algorithms, produce classifications even if there is no discontinuity in the data set, potentially leading to false conclusions about the within‐sample variation. In model‐based clustering, where finite mixture of distributions are fitted, calculating information criteria, such as BIC (Fraley & Raftery, 1998) or integrated complete‐data likelihood criterion (ICL, Biernacki, Celeux, & Govaert, 2000), are the standard way for selecting the best classification. A plethora of methods is available for testing the quality (also called validity or efficiency) of classifications without fitting probability distribution, each applying more or less differently formalized criteria (Handl, Knowles, & Kell, 2005; Milligan & Cooper, 1985; Vendramin, Campello, & Hruschka, 2010). One of the most commonly applied methods for assessing cluster validity is silhouette width (Rousseeuw, 1987), which encompasses two clustering criteria: *separation* (i.e., average distance to the closest other cluster) and *compactness* (i.e., average within‐cluster distance; Handl et al., 2005). It is originally defined for crisp classification but Campello and Hruschka (2006) presented a generalization to fuzzy memberships. Silhouette width is calculated for each object of the classification thus indicating how well they fit into their respective cluster. The cluster‐wise or the global mean of objects can be used to assess the distinctness of specific clusters or the validity of the total classification, respectively, higher means suggesting more efficient classification. Due to the compactness criterion involved as average within‐cluster distance, silhouette prefers spherical cluster shapes (Rousseeuw, 1987); however, in practice clusters can possess different shapes according to their structure in the multidimensional space of the variables. Moreover, each clustering algorithm has its own tendency to produce clusters with certain characteristics, including cluster shape, and evaluating them by validity indices following different shape criteria can bring misleading results (Handl et al., 2005). Hence, in the presence of nonspherical clusters, silhouette width may falsely suggest low classification efficiency. Those indices are more suitable for elongated or irregular cluster shapes which quantify the degree to which objects are assigned to the same cluster as their nearest neighbors, that is, those applying the *connectedness* criterion (Saha & Bandyopadhyay, 2012).

In this paper, we propose a generalization of the silhouette width. Applying the generalized mean, we propose a flexible formula which allows for scaling the sensitivity of the index between connectedness and compactness, thus allowing high values for nonspherical clusters. This enables users to optimize classifications for different cluster shapes depending on the relevance of connectedness versus compactness criteria for the research question. Generalized mean has a parameter (denoted by *p*) that determines the importance of connectedness and compactness. Parameter *p* is analogous to the scale parameter of Hill diversity (Hill, 1973) that determines the importance of rare and common species in determining diversity of a community (In fact, Hill diversity can be regarded as weighted generalized mean of rarity, see Leinster & Cobbold, 2011). The use of the new method is illustrated on artificial point patterns and a widely known real sample data set. Our goal is showing how generalized silhouette width with different *p* parameters evaluate typical classification patterns; at the same time, we do not aim at nominating an optimal *p* parameter value (as neither is there an optimal scaling parameter for Hill diversity) or classification method.

## 2 MATERIALS AND METHODS

### 2.1 The original silhouette width

The original definition of silhouette width according to Rousseeuw (1987) is as follows. Let *i* be a focal object belonging to cluster *A*. Denote by *C* a cluster not containing *i*. *a*(*i*) is defined as the average dissimilarity between *i* and all other objects in *A*, while *c*(*i*,*C*) is the average dissimilarity between *i* and all objects in *C*.

$$b\left(i\right)=\min_{C\neq A}c\left(i,C\right)$$

The silhouette width, *s*(*i*), is defined as:

$$s\left(i\right)=\frac{b\left(i\right)-a\left(i\right)}{\max\left\{a\left(i\right),b\left(i\right)\right\}}$$

*s*(*i*) ranges between −1 and 1. Values near 1 indicate that object *i* is much closer to the other objects in the same cluster than to objects of the closest other cluster, implying a correct classification. If *s*(*i*) is near 0, the correct classification of the focal object is doubtful, thus suggesting intermediate position between two clusters. *s*(*i*) near −1 indicates obvious misclassification. Accordingly, averaging silhouette widths over a cluster gives an assessment of the “goodness” of that cluster, or a sample‐wise average can be used as an index of the validity of the entire classification. Instead of cluster‐wise or sample‐wise averages of *s*(*i*), the number or proportion of objects with positive silhouette width can also be used as validity measures. For a cluster containing a single object, *s*(*i*) takes the arbitrary value 0.

### 2.2 Implementing the generalized mean

Applying the arithmetic mean to calculate average within‐ and between‐cluster distances, as the index was introduced originally (Rousseeuw, 1987), implies that the ideal cluster shape is spherical. However, this preference can be relaxed by choosing other types of means. Generalized mean (also called Hölder or power mean) offers a flexible solution to calculate sample means ranging between minimum and maximum (Cantrell & Weisstein, 2019). Let *X* be a sample of positive real numbers *x*_1_, *x*_2_, …, *x*_n_ and *p* an element of affinely extended real numbers. The generalized mean of degree *p* is as follows:

$$M^{p}\left(x_{1},\ldots x_{n}\right)={\left(\frac{1}{n}\sum_{k=1}^{n}x_{k}^{p}\right)}^{\frac{1}{p}}$$

For *p* = 0 and *p* = |∞| the following exceptions are to be made:

$$M^{0}\left(x_{1},\ldots x_{n}\right)=\lim_{p\to 0}M^{p}\left(x_{1},\ldots x_{n}\right)={\left(\prod_{k=1}^{n}x_{k}\right)}^{\frac{1}{n}}$$

$$M^{-\infty }\left(x_{1},\ldots x_{n}\right)=\lim_{p\to -\infty }M^{p}\left(x_{1},\ldots x_{n}\right)=\min\left(x_{1},\ldots x_{n}\right).$$

$$M^{\infty }\left(x_{1},\ldots x_{n}\right)=\lim_{p\to \infty }M^{p}\left(x_{1},\ldots x_{n}\right)=\max\left(x_{1},\ldots x_{n}\right).$$

The generalized mean takes the values of well‐known summary statistics presented in Table 1. The original version of silhouette width is the special case when within‐ and between‐group average distances are calculated by *p* = 1. By changing the *p* parameter, it is possible to emphasize lower or higher distances in the calculation of means. The lower the *p* parameter is, the more importance is attributed to objects in close proximity, while the effect of farther neighbor objects (including outliers) is reduced. In this way, the criteria of compactness are gradually replaced by connectedness and clusters with irregular or elongated shape can also be considered “good”. At *p* = −∞, a classification is ideal if each object is assigned to the same cluster as the most similar other object in the sample. This procedure follows the logic of single linkage clustering, while the original version making use of arithmetic averages followed the logic of average linkage. In contrast, when *p* > 1, the compactness criterion is attributed higher weight; thus, the preference toward spherical clusters is further increased and the effect of outliers on the overall classification should become more significant. At *p* = +∞, the clustering criteria of complete linkage are applied.

**Table 1:** Special cases of the generalized mean

*p*	Descriptive statistic
−∞	Minimum
−1	Harmonic mean
0	Geometric mean
1	Arithmetic mean
2	Quadratic mean (root‐mean‐square)
+∞	Maximum

### 2.3 Data sets and tests

We test the performance of the generalized mean with different parameterization on artificial point patterns and a well‐known public data set.

Artificial data sets containing 100 objects and two variables were generated. The data sets represented data structures some of which were also applied by Podani (2000) for the illustration of the behavior of different clustering methods: (a) completely random point pattern without true clustered structure, points on the two sides of the plane are assigned to different clusters (low separation, low compactness); (b) two clusters with few transitional elements between them (moderate separation and compactness); (c) two distinct point aggregations corresponding to two clusters (high separation, high compactness); (d) two overlapping clusters, both containing point duplicates with a little offset, thus each point has a “pair” (or close neighbor) belonging to the same cluster (low separation, high compactness, high local connectedness); (e) the same point pattern but members of pairs belong to different clusters (low separation, high compactness, low local connectedness); (f) two clusters of elongated shape running parallel (high separation, low compactness); (g) two well‐separated clusters of unequal size (20 vs. 80 points) and spread (high separation, high compactness, unequal size); (h) two concentric clusters (high separation, different compactness, special spatial arrangement).

The Iris data set was originally published by Fisher (1936). It contains morphological measurements of 150 individuals of *Iris setosa*, *Iris virginica*, and *Iris versicolor*, 50 individuals each. *Iris setosa* is morphometrically distinctly separated from the other two, while *I. virginica* and *I. versicolor* differ rather gradually. The original data set contained four variables, from which we used only two, sepal length and petal length, for the possibility of plotting the total variation in two dimensions. Species assignment was used as a priori classification. Data were accessed from the vegan (Oksanen et al., 2018) package of the R software (R Core Team, 2017); then, variables were standardized to mean = 0 and standard deviation = 1.

On these data sets, generalized silhouette widths with different *p* parameter values were calculated using the a priori classifications. *p* parameters were selected for the tests with the aim of representing the descriptive statistics which are special cases of the generalized mean (Table 1) and being spread evenly across values near zero. Patterns of misclassified objects (i.e., objects with negative silhouette width) on the point scatters were assessed visually. Overall classification quality was measured by misclassification rate (MR; the number of misclassified objects in the sample divided by the total number of objects) and mean silhouette width (MSW; the sample‐wise mean of *s*(*i*)).

We evaluated also the performance of different classification methods in the view of the generalized silhouette width. For this purpose, we used a two‐dimensional random point pattern of 1,000 points because we supposed that in the lack of true cluster structure the inherent characteristics of the methods will determine classification the most. We classified this data set using single linkage, group average, complete linkage, and beta flexible (with beta = −0.25) methods. Silhouette width with different *p* parameters was calculated at each group number of the hierarchical classifications between 2 and 20; then, mean silhouette widths were compared across group numbers, *p* parameters and classification methods. Given the nonclustered structure of this data set, we do not expect a peak in the change of MSW which would indicate an “optimal” cluster level.

Computations were carried out by the R software (R Core Team, 2017) using the cluster package (Maechler, Rousseeuw, Struyf, Hubert, & Hornik, 2018). Program codes for silhouette width using generalized mean and for generating artificial data sets are available in the Supporting Information.

## 3 RESULTS

**Figure 1:**
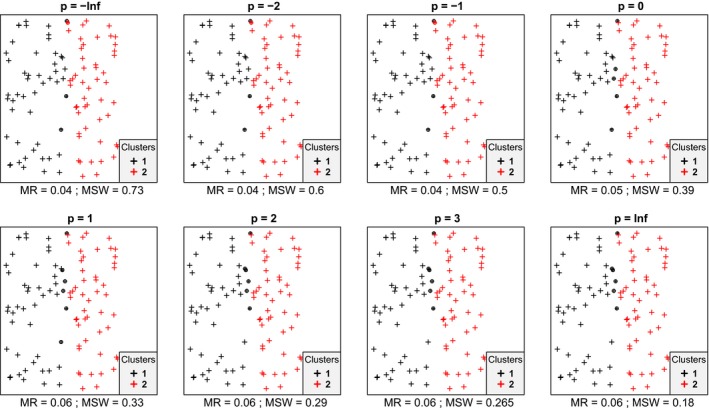
Silhouette width patterns of objects grouped into two clusters with low separation and low compactness. MR, misclassification rate; MSW, mean silhouette width; misclassified objects are circled

**Figure 2:**
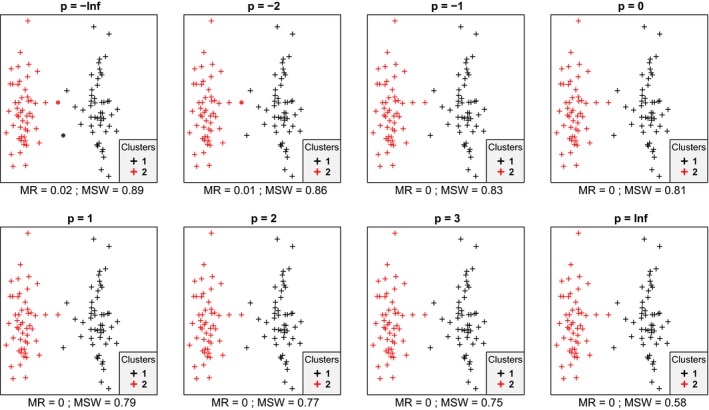
Silhouette width patterns of objects grouped into two clusters with moderate separation and moderate compactness. MR, misclassification rate; MSW, mean silhouette width; misclassified objects are circled

**Figure 3:**
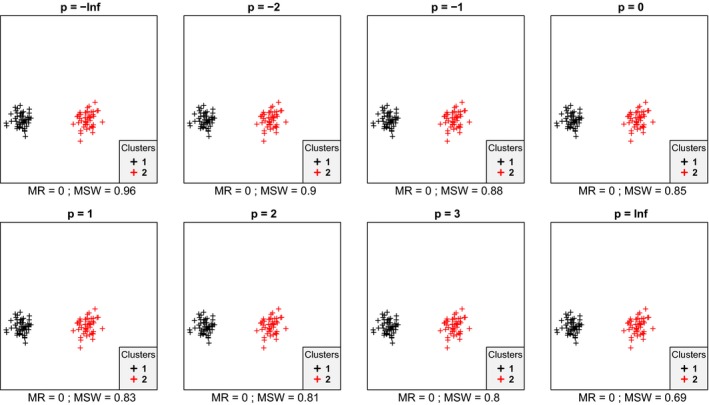
Silhouette width patterns of objects grouped into two clusters with high separation and high compactness. MR, misclassification rate; MSW, mean silhouette width; misclassified objects are circled

**Figure 4:**
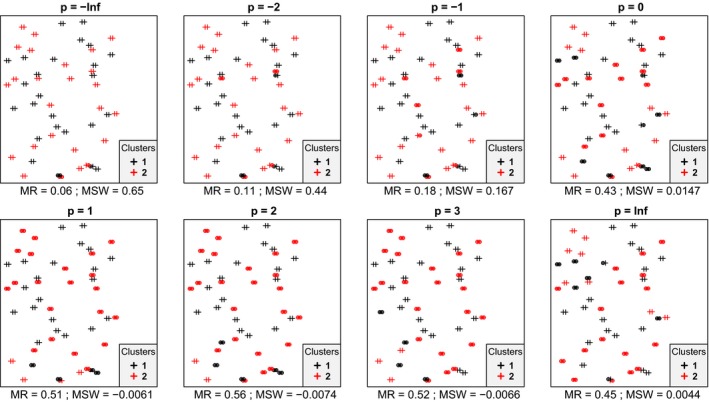
Silhouette width patterns of objects grouped into two overlapping clusters, both containing point duplicates with a little offset; thus, each point has a “pair” (or close neighbor) belonging to the same cluster (low separation, high compactness, high local connectedness). MR, misclassification rate; MSW, mean silhouette width; misclassified objects are circled

**Figure 5:**
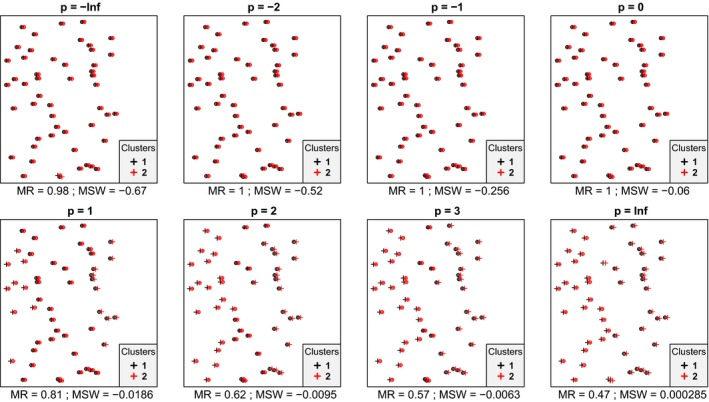
Silhouette width patterns of objects grouped into two overlapping clusters, both containing point duplicates with a little offset; thus, each point has a “pair” (or close neighbor) belonging to the other cluster (low separation, high compactness, low local connectedness). MR, misclassification rate; MSW, mean silhouette width; misclassified objects are circled

**Figure 6:**
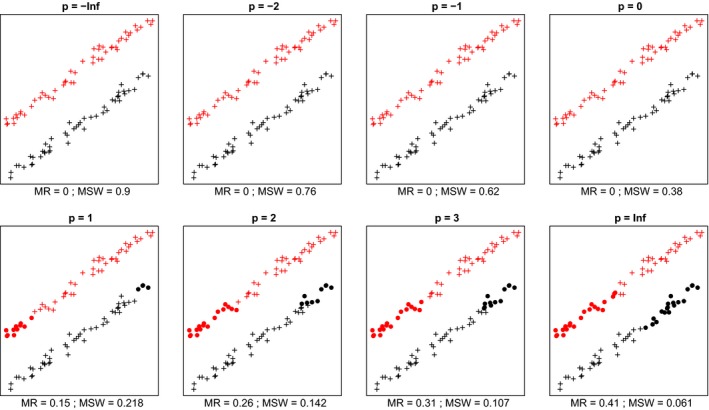
Silhouette width patterns of objects grouped into two, parallely situated clusters with high separation and low compactness. MR, misclassification rate; MSW, mean silhouette width; misclassified objects are circled

**Figure 7:**
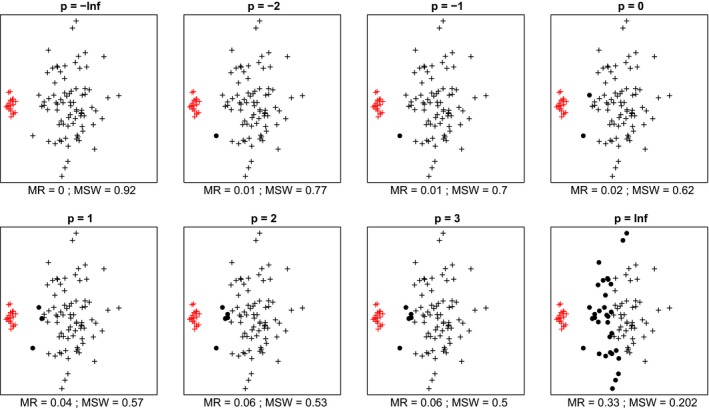
Silhouette width patterns of objects in two aggregates grouped into two clusters with high separation, high compactness and different size. MR, misclassification rate; MSW, mean silhouette width; misclassified objects are circled

**Figure 8:**
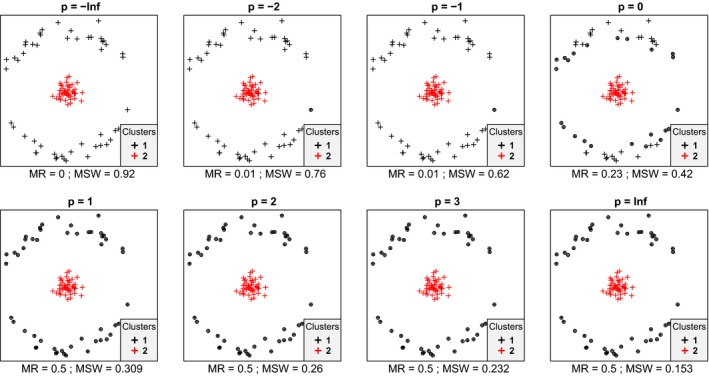
Misclassification patterns of objects grouped into two concentric clusters with good separation—an outer one with low compactness and an inner one with high compactness. MR, misclassification rate; MSW, mean silhouette width; misclassified objects are circled

In most cases, we inspected, within each data set mean silhouette width (MSW) decreased with increasing *p*. With artificial data, when the point pattern was random, for *p* parameter values up to zero there were four or five misclassified objects, while for higher *p* there were six ones (Figure 1). Despite the low misclassification rate, MSW decreased from 0.73 at *p* = −∞ to 0.18 at *p* = ∞. Misclassified plots were situated near the border between the two clusters. When the separation and compactness were moderate (Figure 2), for *p* = −∞ and *p* = −2 there were two and one misclassified objects, respectively, otherwise all plots were correctly clustered with higher *p* parameter values. There were no misclassifications at all when points were clustered into two well‐separated aggregations (Figure 3); however, MSW decreased from 0.96 to 0.69 with increasing *p*. In case of overlapping clusters with duplicate offset pairs of points, misclassification rate increased from 0.06 to 0.45, while MSW decreased from 0.65 to 0.0147 as *p* was increased from −∞ to 0 (Figure 4). Between *p* = 1 and *p* = ∞ MR showed no clear trend, MR took values near 0.5, while MSW varied near 0. Uniquely, *p* = 2 showed the highest MR, 0.56. We could not recognize clear pattern in the occurrence of misclassified points. The only case when MR decreased (from 0.98 to 0.47) and MSW increased (from −0.67 to −0.000285) with increasing *p* was when the pairs of points belonged to different clusters (Figure 5). Interestingly, with *p* = 2 and higher, both classifications (Figures 4 and 5) seemed similarly efficient, both for MR (varying near 0.5) and MSW (varying around 0). At negative *p* parameters, most points were misclassified giving a uniform pattern. From *p* = 1 or higher misclassifications were becoming restricted to one side of the scatter for each cluster with decreasing overlap in the middle of the point cloud. At *p* = ∞ misclassified objects of cluster 1 were located on the right‐hand side of the plot, while those of cluster 2 on the left‐hand side. With parallel groups, all objects were considered correctly classified with *p* < 1 (Figure 6). From *p* = 1 the MR increased from 0.15 to as high as 0.41 at *p* = ∞. At *p* = −∞ MSW was 0.9, then gradually decreased until reaching 0.061 at *p* = ∞. Objects in marginal position in the point clouds tended to be identified as misclassified. When two, well‐separated and compact groups were of different sizes, MR and MSW decreased as *p* increased (Figure 7). With *p* = −∞, there was no misclassification, and MSW was 0.92. With increasing *p*, misclassified objects appeared gradually in the larger cluster near the border of the two clusters but they were not abundant until *p* = 3. However, with *p* = ∞ as high as 33% of all objects were indicated misclassified, all belonging to the larger group, and MSW were 0.202. With concentric groups, the inner, compact group was considered perfect regardless the *p* parameter (Figure 8). However, the assessment of the outer group varied greatly. With *p* = −∞ all objects were deemed correctly classified. As *p* raised, the number of misclassified objects in the outer group increased, too. With *p* = 0, misclassified plots gave 23% of the total data set which means 46% of the outer group. From *p* = 1 and higher, all objects in the outer group were considered misclassified; thus, the data set consisted of a perfect and a totally bad cluster together giving 50% correct classification rate. Along the gradient in the parameter value, MSW decreased from 0.92 (*p* = −∞) to 0.153 (*p* = ∞).

**Figure 9:**
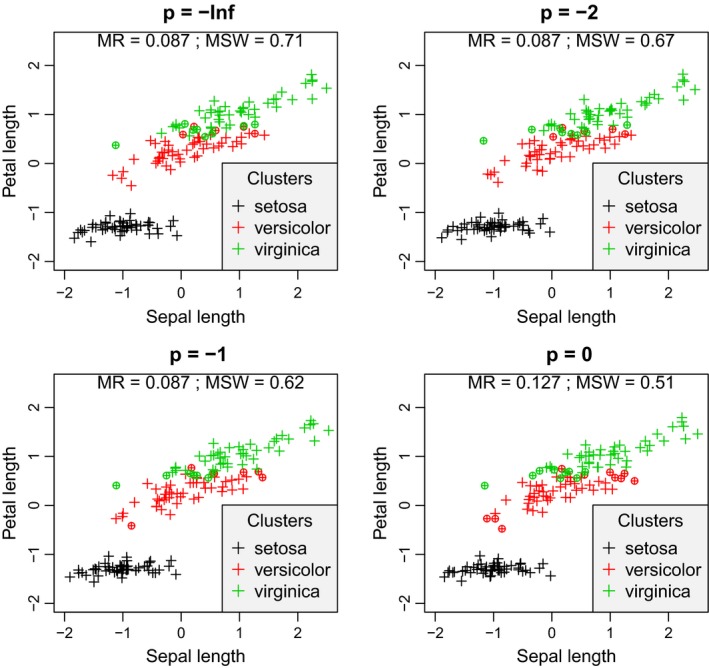
Silhouette width patterns of the Iris data set using sepal length and petal length variables after standardization to mean = 0 and standard deviation = 1 with *p* ranging from −∞ to 0. MR, misclassification rate; MSW, mean silhouette width; misclassified objects are circled

**Figure 10:**
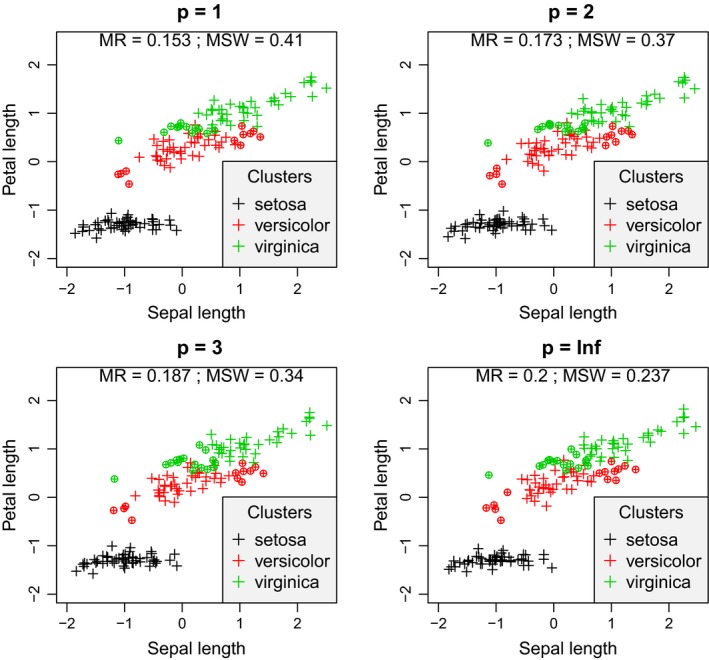
Silhouette width patterns of the Iris data set using sepal length and petal length variables after standardization to mean = 0 and standard deviation = 1 with *p* ranging from 1 to +∞. MR, misclassification rate; MSW, mean silhouette width; misclassified objects are circled

Similarly to the simulated data, with the Iris data set, misclassification rate increased with increasing *p* parameter (Figures 9 & 10). The minimum was 0.087 with *p* < 0, and the maximum was 0.200 at *p* = ∞. MSW decreased from 0.71 to 0.237. *Iris setosa* was perfectly separated from the other two groups since none of its members obtained negative silhouette width with any value of *p*. At the area where *I. versicolor* and *I. virginica* overlap, there were misclassified objects according to all values of *p*. However, with increasing *p*, *I. versicolor* individuals at the opposite end of the point cloud of the cluster, that is, closer to points of *I. setosa*, also tended to seem misclassified.

**Figure 11:**
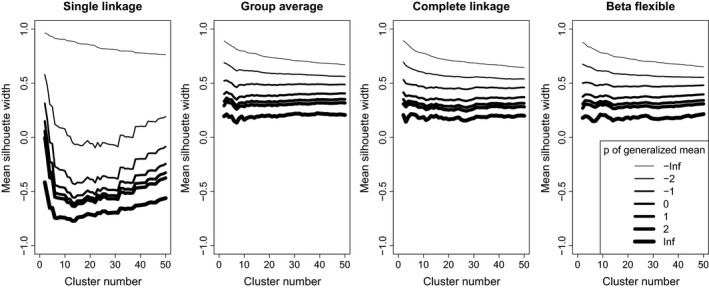
Comparison of mean silhouette widths calculated with different *p* parameter values on classifications with different methods and cluster numbers—a comparison between *p* parameter values separating the effect of classification methods

**Figure 12:**
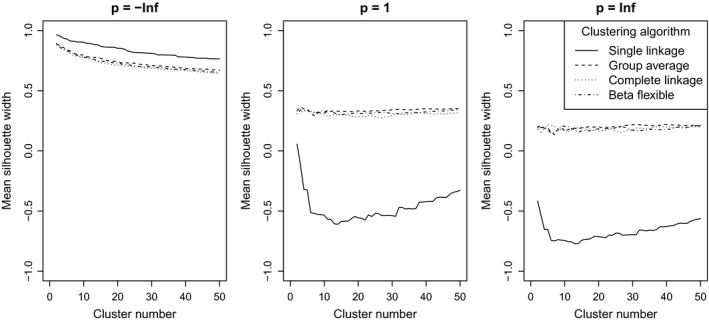
Comparison of mean silhouette widths calculated with different *p* parameter values on classifications with different methods and cluster numbers—a comparison between classification methods, separating the effect of *p* parameter values

With all classification methods on the random data, mean silhouette width decreased with increasing the *p* parameter (Figure 11). Using single linkage and *p* = −∞, MSW decreased monotonically with increasing number of clusters, while with higher *p*, it first decreased until a minimum between 10 and 30 clusters then increased with the number of clusters. With group average, complete linkage, and beta flexible low (typically −∞ and −2) *p* parameters resulted in MSW curves decreasing monotonically, while higher *p* parameters did not show clear trend; although, local peaks and “valleys” were often visible near 3 to 10 clusters, and with higher *p* parameter values, MSW tended to increase toward high number of clusters. Nevertheless, the effect of changing the *p* parameter was significantly stronger on MSW when the data set was classified by the single linkage method than with the other two. When methods were compared, with *p* = −∞, single linkage obtained the highest MSW, followed by group average, and finally complete linkage and beta flexible—although, the latter three performed very similarly (Figure 12). With *p* = 1 (i.e., the original version of silhouette width) and *p* = ∞, group average, complete linkage, and beta flexible performed very similarly, while single linkage obtained by far the lowest silhouette widths.

## 4 DISCUSSION AND CONCLUSIONS

The results supported our expectation about the behavior of the silhouette method using the generalized mean. Both artificial data and the Iris data set showed that cluster compactness plays a decreasingly significant role in the assessment of classification validity with decreasing *p* parameter value. With *p* << 0, clusters are assessed mainly on the basis of connectedness and separation criterion. In the extreme case (*p* = −∞), it means the relativized difference between the minimal distances of objects belonging to the same cluster versus minimal distances of objects belonging to the closest other cluster, while distances from other members of the same and the neighbor cluster are completely disregarded. As we increase the *p* parameter, more importance is attributed to more distant objects within and between clusters, that is, to the compactness criterion.

In most cases, mean silhouette width decreased and misclassification rate increased with increasing *p* parameter value. In other words, these classifications tended to seem decreasingly efficient as the compactness criterion was attributed more and more importance. The only exception was Figure 5 which illustrated an obviously inefficient classification. Notably, across all tests, MSW with *p* = −∞ ranged from −0.67 to 0.96, while with *p* = ∞, this interval was much narrower, between −0.000285 and 0.69. Misclassification rate seemed not less sensitive to the change of *p* parameter since it varied between 0 and 0.98 with *p* = −∞ and between 0 and 0.5 with *p* = ∞. Conclusively, the relationship between MSW or MR and the *p* parameter value is highly dependent on the data set and on the classification but with lower *p* MSW and MR vary on broader range. Therefore, special caution is advised if silhouette‐based validity indices obtained with different *p* parameters are compared. Probably such comparisons are valid only if, instead of the raw MSW or MR, their standardized difference from the expected value is used (Handl et al., 2005). Such an expected value should be estimated using randomization procedures following an appropriate null model.

With different values of the *p* parameter silhouette width considers different clustering strategies effective. As it was expected, low *p* parameter values prefer algorithms which disregard cluster compactness, for example, single linkage, while with high *p*, procedures resulting in spherical clusters (e.g., group average, complete linkage, beta flexible) are deemed better. In the comparison of classification methods in the view of the generalized silhouette width, group average, complete linkage, and beta flexible behaved similarly efficiently across different *p* parameter values and cluster numbers. One of its potential explanations is that in the case of sequential clustering methods, including those applied here, earlier agglomerative steps constrain later ones; therefore, the final classification solution may not be the best in terms of silhouette width using the respective value for *p*.

In our analyses, we showed examples which illustrate some characteristic patterns frequently occurring in data analyses or revealing important properties of the silhouette index. Besides separation and compactness, there are several other properties, like the numbers of clusters, shape, size, and orientation of the point aggregations, which can have serious influence on its performance. In the future, more specific testing of these properties in a factorial design could offer a lengthy but more specific assessment of the behavior of generalized silhouette width.

There are many other cluster validation indices that combine cluster separation and compactness (Handl et al., 2005; Vendramin et al., 2010); however, silhouette width is the only one that evaluates individual objects. Generalized mean instead of arithmetic mean (or minimum or maximum) could be used in other indices combining the separation and the compactness criteria. Similar examples are already shown by Bezdek and Pal (1998) for the generalization of the Dunn index.

Clusters of natural objects frequently show nonspherical shapes in the multidimensional space of the analysis. In such cases, a cluster validity measure with a preference toward spherical shape can evaluate cluster quality too rigorously. When it is not reasonable to expect spherical (i.e., very compact) clusters but only their connectedness and separation are relevant, setting *p* to negative values to assess the fit of objects into the classification can be a solution. However, care should be taken with negative *p* parameters, too. As it was shown on Figure 4, overemphasizing the local‐scale connectedness criterion completely disregards global shape and position of clusters leading to unintuitive or questionable result. On Figure 4, objects had a close neighbor belonging to the same cluster, although there was a high overlap between the scatters of the two clusters on larger scale. This classification was found very effective with negative *p* parameter values but very dubious with positive *p*. Positive *p* parameter values gave similar results on data sets 4 and five despite their fine‐scale differences. To avoid such pitfalls, we advise to calculate silhouette width with different values of *p*, which could be a logically similar procedure to calculating diversity profiles using scalable diversity measures (Tóthmérész, 1995). In this way, a new dimension of methodological decisions referring to cluster compactness can be involved in the assessment of classifications (Lengyel, Landucci, Mucina, Tsakalos, & Botta‐Dukát, 2018). However, we recall that raw silhouette widths with different parameterization may not be directly comparable since with lower *p* parameters silhouette widths vary on broader range. Hence, curves of average silhouette width with different *p* parameters along number of clusters should be viewed as coming from different indices which are ordered by sensitivity to compactness, and no “optimal *p* parameter value” should be sought for empirically. When it is necessary to choose a single *p* for pragmatic reasons, the choice should be primarily driven by the researcher's view about the biological relevance of compactness versus connectedness to the actual research question.

Despite nonsphericity, when the distribution of variables within clusters is known, clustering objects and evaluating the classification are possible in a model‐based framework. Mixture models, especially Gaussian mixture models, are now widely used for classification and subsequent cluster evaluation (Banfield & Raftery, 1993; McNicholas, 2016); while in ecology and evolution, they are not common yet (but see Dantas, Hirota, Oliveira, & Pausas, 2016; Perry, Miller, Lamont, & Enright, 2016). However, distributional properties, including shape, of clusters are often not known before the analysis, and the application of an unsuitable model for defining clusters and/or evaluating the fit of objects into the classification may give misleading results. By contrast, being a dissimilarity‐based method, silhouette width can be used for evaluating already existing classifications with no assumption on the distribution of variables. However, it requires cluster memberships and a dissimilarity matrix of objects, thus implying decisions on the classification algorithm and the resemblance measure. Depending on which assumptions on sample properties are likely reasonable and which methodological decisions seem straightforward, investigators should decide between model‐based and dissimilarity‐based approaches. Having an already existing classification and reliable dissimilarity estimations in hand, we recommend generalized silhouette width for cluster evaluation.

Future research should explore the possibility of adapting the generalized mean into other developments of the silhouette width (e.g., for fuzzy classifications, Campello & Hruschka, 2006) and applicability as a classification criterion (e.g., in the OPTSIL algorithm, Roberts, 2015).

## CONFLICT OF INTEREST

None declared.

## AUTHOR CONTRIBUTIONS

A.L. developed the idea and the methodology, wrote the scripts, conducted data analysis, and wrote the manuscript; Z.B.D. developed the idea, reviewed literature, commented on the results, and improved the manuscript.

## Data Availability

Scripts for calculating generalized mean and generating specific point patterns are enclosed in the Supporting Information. Iris data set is available from the vegan package of R.
