# Supplementary material for: Silhouette width using generalized mean—A flexible method for assessing clustering efficiency
Source: Ecol Evol. 2019 Nov 19;9(23):13231–43. doi: 10.1002/ece3.5774 (PMC6912895; doi:10.1002/ece3.5774)
Supplement: Supplementary file 1 [file ECE3-9-13231-s001.docx]

Supporting Information for the manuscript

**Attila Lengyel & Zoltán Botta-Dukát: Silhouette width using generalized mean – a flexible method for assessing clustering efficiency**

to be published in the journal Ecology and Evolution

**R functions**

genmean

Returns a generalized mean of a numeric vector.

x: the sample as a numeric vector

p: the power as a single value

genmean<-function(x,p) {

x<-x[!is.na(x)]

n<-length(x)

M<-((1/n)*(sum(x^p)))^(1/p)

if(p==-Inf) M<-min(x)

if(p==-1) M<-(sum(x^-1)/length(x))^-1

if(p==0) {x[x==0]<-1e-10; M<-exp(mean(log(x)))}

if(p==Inf) M<-max(x)

return(M)

}

mdist

Calculates generalized mean distance between a single object and other objects which are classified into groups. Returns a vector of length k where k is the number of clusters.

d: a vector of distances/dissimilarities between the focal object and all other objects in the sample. d should not contain the distance of focal object from itself, which is 0.

gr: clustering of object from which distances are calculated. Can be a vector of integers or a factor. length(gr) should equal length(d).

p: the power of generalized mean.

mdist<-function(d,gr,p) {

GR<-unique(gr)

k<-length(GR)

mean.d<-vector('numeric')

for(i in 1:k) {

mean.d[i]<-genmean(d[gr==GR[i]], p=p)

}

return(mean.d)

}

silgen

Calculates silhouette width with generalized mean for distance matrix with a classification. Returns a matrix with observations as rows, silhouette widths in the first column, original classification in the second column, nearest neighbour clusters in the third column.

d: contains pair-wise distances/dissimilarities, can be either a matrix or an objects of class dist.

gr: grouping of objects, a vector of integers or a factor.

p: the power.

silgen<-function(d,gr,p) {

di<-as.matrix(d)

diag(di)<-NA

GR<-unique(gr)

N<-nrow(di)

Do<-matrix(NA, nrow=N, 3)

i<-1

while(i<=N) {

Dvec<-mdist(d=di[i,],gr=gr,p=p)

K<-which(GR==gr[i])

if(sum(gr==gr[i])>1) {a<-Dvec[K]

} else {a<-0}

Kx<-which(Dvec==min(Dvec[GR!=gr[i]]))[1]

b<-Dvec[Kx]

do1<-ifelse(a==0 & b==0, do1<-0, do1<-(b-a)/max(a,b))

Do[i,1]<-do1

Do[i,2]<-gr[i]

Do[i,3]<-GR[Kx]

i<-i+1

}

colnames(Do)<-c("Width","Original","Neighbour")

return(Do)

}

**Codes for generating artificial data**

N<-100 #the number of points

Random point scatter with two clusters

x<-sort(runif(N, 0,10))

y<-runif(N,0,10)

A<-cbind(x,y)

plot(A)

Points in two well-separated aggregations

x1<-rnorm(N/2,0,1)

x2<-rnorm(N/2,10,1)

x<-c(x1,x2)

y<-rnorm(N)

B<-cbind(x,y)

plot(B)

Points in two groups with transition

C1<-matrix(c(runif(0.1*N,-0.5,0.5),rnorm(0.1*N,0,0.05)),nrow=0.1*N, byrow=F)

C2<-matrix(c(rnorm(0.45*N,-0.5,0.1),rnorm(0.45*N,0,0.1)),nrow=0.45*N, byrow=F)

C3<-matrix(c(rnorm(0.45*N,0.5,0.1),rnorm(0.45*N,0,0.1)),nrow=0.45*N, byrow=F)

C<-rbind(C1,C2,C3)

plot(C)

Offset points in two overlapping clusters

x1<-runif(N/2)

y1<-runif(N/2)

x2<-x1+0.02

y2<-y1

x<-c(x1,x2)

y<-c(y1,y2)

H<-cbind(x,y)

plot(H)

Parallel groups

di<-runif(N, 0, 20)

plus<-sort(sample(c(-3, 3), N, replace=T))

Di<-matrix(rnorm(N,c(0.6*di+plus), sd=0.5), nrow=N, byrow=F)

Di<-cbind(di, Di)

plot(Di)

Concentric groups

x1<-runif(N/2,-1,1)

y1<-sqrt(1-x1^2)*sample(c(-1,1),N/2,replace=T)

y1<-rnorm(N/2,y1,0.1)

E1<-cbind(x1,y1)

x2<-rnorm(N/2,0,0.1)

y2<-rnorm(N/2,0,0.1)

E2<-cbind(x2,y2)

E<-rbind(E1,E2)

plot(E)

Compact groups of different sizes

x<-c(rnorm(20,0,0.3),rnorm(80,5,1.5))

y<-c(rnorm(20,0,0.3),rnorm(80,0,1.5))

G<-cbind(x,y)

plot(G)

Standard analysis of a custom data set

ord<-A #with the example of data set A (random point, two groups)

dist1<-dist(ord)

groups<-as.factor(c(rep(1,N/2),rep(2,N/2)))

PP<-c(-Inf, -2, -1, 0, 1, 2, 3, Inf) #setting values for the *p* parameter

par(mfrow=c(2,4), mar=c(3,2,2,1))

for(i in 1:8) {

SF<-silgen(d=dist1,gr=groups,p=PP[i])

ppch<-rep(3,nrow(SF))

ppch[SF[,1]<0]<-10

mis<-sum(SF[,1]<0)/N

mean.width<-zapsmall(mean(SF[,1]),2)

plot(ord,col=as.numeric(groups), cex=1, main=paste("p=",PP[i]), pch=ppch, yaxt="n", xaxt="n", xlab=NA, ylab=NA, cex.main=1.5, cex.sub=1.2, xlim=c(min(ord[,1]),max(ord[,1])*1.3))

mtext(paste("MR=",mis, "; MSW=", mean.width),side=1, line=0.5)

legend("bottomright",legend=levels(groups),col=1:3, pch=3, bg="grey95", cex=1.2, box.lwd=1, title="Clusters", pt.lwd=2, text.font=2)

}
